# Experimental Investigation of High-Cycle Compressive Fatigue Performance of C80 High-Strength Concrete

**DOI:** 10.3390/ma19050958

**Published:** 2026-03-02

**Authors:** Laiyuan Qin, Jia Fu, Mingyi Zhang, Ruiquan Zhou, Weifeng Tao, Zhiqiang Wan, Pengfei Wang

**Affiliations:** 1 School of Mechanics and Transportation Engineering, Northwestern Polytechnical University, Xi’an 710129, China; qinlaiyuanty@163.com (L.Q.); wanzhiqiang@nwpu.edu.cn (Z.W.); 2 Shanghai Electric Wind Power Group Co., Ltd., Shanghai 200233, China; zhourq@shanghai-electric.com; 3 CFHI (Heilongjiang) Wind Power Hybrid Tower Co., Ltd., Qiqihar 161042, China; 4 School of Civil Engineering, Xijing University, Xi’an 710123, China; taoweifeng2048@126.com; 5 Center of Materials Science and Optoelectronics Engineering, University of Chinese Academy of Sciences, Beijing 100049, China; pfwang@opt.ac.cn

**Keywords:** C80 concrete, high-cycle fatigue life, *S–N* curve, strain evolution, stiffness degradation

## Abstract

With the height of wind turbine towers increasing, the high-cycle fatigue performance of high-strength concrete has become important for structural design. This study systematically investigates the fatigue life, strain evolution, and stiffness degradation of C80 concrete under constant-amplitude cyclic compressive loading for a maximum stress level ranging from 0.70 to 0.90 and a minimum stress level of 0.10. Based on experimental data, *S–N* curves are obtained, and a prediction model of fatigue life and stiffness degradation is developed. The results reveal that fatigue strain evolves through three stages and that the second stage accounts for more than 90% of the overall fatigue life, exhibiting linear growth over time. The final strain in the second stage is very close to that in static compression tests, indicating the uniqueness of fatigue strain. In addition, the final strain in the second stage provides a better prediction of fatigue life than an *S–N* curve and facilitates real-time fatigue life prediction. Meanwhile, the stiffness degradation model more accurately simulates the stiffness degradation process of C80 concrete under fatigue load, laying a foundation for further finite element analysis of fatigue. This study addresses the gap in fatigue life prediction and stiffness degradation modeling for C80 concrete under high-cycle fatigue load, providing a valuable reference for designing safe and durable high-strength concrete structures such as wind turbine towers.

## 1. Introduction

Traditionally, wind turbines were built to a convenient height below 100 m; however, this has been exceeded recently with the rapid development of the global economy. Higher wind turbine towers are being built to increase the sweeping area of the turbine blades and to reach high-speed wind at higher altitudes [1,2]. As a result, greater demands are placed on the bearing capacity and performance of tower structures. However, increased tower height significantly increases self-weight and structural flexibility, thus amplifying dynamic responses and increasing fatigue load under wind excitation [3].

There are a variety of wind turbine towers, including steel, concrete, and hybrid steel–concrete towers; among these, the latter are the most widely used in practice due to their economical cost, strong stiffness, and high strength. Currently, for hybrid towers to possess sufficient strength, concrete of grade C80 [4] or higher is necessary. While significant progress has been made in understanding the chemical phases of cementitious materials to achieve its sustainable synthesis [5,6], the long-term macroscopic fatigue response of C80 concrete under millions of cycles remains a critical challenge in engineering design. Wind turbine towers must have a service life of at least 20 years, during which time they are anticipated to experience approximately 10^8^ to 10^9^ cycles of periodic loading [7], far exceeding the fatigue demands on conventional building structures.

Given the increase in tower heights and the corresponding high-cycle fatigue demands on high-strength concrete, understanding this material’s fatigue performance has become crucial. In early studies, Aas-Jakobsen, Tepfers, and others [8,9,10] conducted extensive experiments to investigate the fatigue life of normal-strength concrete (C20–C40), establishing empirical *S–N* curve models that gained widespread acceptance and laid the foundation for subsequent research. Subsequently, Holmen [11] conducted in-depth studies on the deformation and fatigue performance of C40 concrete under constant- and variable-amplitude compressive cyclic stresses. Bennett [12] and Lemaitre [13] investigated the stiffness degradation behavior of concrete under fatigue load and derived corresponding empirical formulas. Furthermore, Buyukozturk et al. [14] and Yin et al. [15] explored the multiaxial fatigue performance of concrete. These findings provided critical insights for improving the fatigue strength of normal-strength concrete.

With increasing engineering demands, fatigue performance studies have progressively extended to high-strength concrete (C60 and above). Oneschkow [16] investigated the fatigue life of C80 concrete by varying the maximum stress level, achieving up to 2 × 10^6^ cycles. Chen [17] investigated the variations in peak stress, peak strain, and elastic modulus of C60 and C45 specimens with diameters ranging from 150 to 460 mm under fatigue loading, and based on the experimental results, a peak stress–strain model considering size effects was proposed. Basaldella [18] studied the fatigue performance of high-strength (C80) and ultra-high-strength concrete (C130), revealing the influence of increased material strength on fatigue damage development, though the study was primarily limited to fatigue lives below 1 × 10^5^ cycles. While these studies established the initial framework for high-strength concrete (HSC), a significant gap in the literature remains, with existing experimental data often limited to the “low-cycle” or “medium-cycle” stage.

Analyses of concrete fatigue are costly and time-consuming, and many scholars have conducted numerical simulations in an effort to predict the damage evolution and fatigue life of concrete. Over the past two decades, with rapid advances in computational capability and modeling algorithms, finite element methods (FEMs) have increasingly been used in research work.

Hordijk [19] proposed a comprehensive cohesive crack model and integrated it with finite element analysis, thereby enabling the numerical simulation of concrete fatigue behavior and opening a new avenue for research in this field. Dobromil et al. [20] proposed a material model for FEM-based simulation of fatigue crack propagation in concrete using *S–N* curves. By converting *S–N* data into material damage parameters, their model effectively simulates damage and crack growth under high-cycle fatigue conditions. Zou et al. [21] develop a three-dimensional finite element model in ABAQUS to simulate the fatigue behavior of side-bonded CFRP-strengthened reinforced concrete (RC) beams. The model is experimentally validated and accurately predicts fatigue life, failure modes, and interfacial damage evolution. In addition, a simplified fatigue life prediction formula is proposed to support practical engineering applications. However, the efficacy of these numerical models heavily relies on the accuracy of materials’ constitutive parameters. Without high-cycle experimental data specifically for C80, researchers are often forced to extrapolate parameters from normal-strength concrete (NSC), which may lead to significant inaccuracies in predictions of the long-term structural integrity of hybrid towers.

To bridge this gap and provide more reliable data for engineering design, this study extends the experimental boundary for C80 concrete to 10^7^ cycles, significantly surpassing the cycle counts in previous research, such as that of Basaldella [18]. This study assesses the high-cycle fatigue behaviors of C80 concrete under cyclic compressive loading by investigating fatigue life up to 10^7^ cycles. While the total design life of wind turbine towers extends to the gigacycle regime, experimental data in the 10^6^–10^7^ range is a critical prerequisite for establishing the fatigue strength of high-strength concrete and calibrating *S-N* curves used for longer-life extrapolations. Furthermore, the regressive formulas developed in this study serve as a direct extension and refinement of the classical *S-N* models proposed by Aas-Jakobsen [8] and Holmen [11], specifically tailored for C80 high-strength applications. The results are intended to support the design and safety assessment of concrete wind turbine towers and to provide the necessary empirical foundation for further numerical simulations using finite element methods, as proposed by Hordijk [19] and Zou [21].

## 2. Experiment

### 2.1. Materials and Specimen Preparation

A high-strength concrete with a target strength grade of C80 was used in this study. All specimens were cast using a single concrete mix design to ensure consistency in material properties throughout the experiment. The concrete mixture consisted of Portland cement, fly ash, ground granulated blast furnace slag (GGBS), fine (sand) and coarse aggregate, water, and a polycarboxylate-based high-range water-reducing admixture. The cement used was P·II 52.5 Portland cement supplied by Shanghai Tunnel Engineering Co., Ltd. Component Branch (Shanghai, China). The fly ash was classified as Class II, and the GGBS had a specific surface area grade of S95. Natural medium sand was used as the fine aggregate, while crushed limestone with a nominal size range of 10–20 mm and continuous grading was adopted as the coarse aggregate. The concrete mixtures are shown in Table 1. The water-to-binder ratio (w/b) of the mixture was approximately 0.26. Both cubic and prismatic specimens were cast in molds, and after a standard 28-day curing period [4], the cubic compressive strength was measured at 81.3 MPa, satisfying the requirements for C80-grade concrete.

According to the Chinese standard GB/T 50082-2009, “Standard for Test Methods of Long-Term Performance and Durability of Ordinary Concrete” [22], the standard specimens in the concrete fatigue experiment were cuboids with dimensions of 100 mm × 100 mm × 300 mm, while the standard specimens employed in the concrete strength tests were cube specimens with a side length of 150 mm, which were used to determine the concrete strength grade. All specimens were cast on the same day, and after demolding, they were cured in a standard curing room with a relative humidity of 99% and a temperature of 20.5 ± 1 °C for 28 days. They were subsequently stored at room temperature (20 ± 5 °C) until they reached an age of 90 days. A total of 30 prismatic specimens were used in the experiments, among which 7, labeled Z-1 to Z-7, were used to determine the axial compressive strength *f*_c_, while the remaining 23 were used for the fatigue tests, as shown in Table 2. The fatigue specimens were designated as F-*i*-*j*, where *i* represents the test group (F-1 to F-5) and *j* indicates the specimen number within that group.

### 2.2. Experimental Procedure

#### 2.2.1. Test on Axial Compressive Strength

In accordance with [22], axial compressive strength tests on prismatic specimens were conducted after 90 days of curing. The load was applied at a constant loading rate of 0.8 MPa/s to determine the stress levels for the compressive fatigue tests, with the latter defined as the ratio of the fatigue loading to the axial compressive strength. The calculation formula is as follows:

(1) $$S_{\max}=\frac{\sigma_{\max}}{f_{c}}$$

(2) $$S_{\min}=\frac{\sigma_{\min}}{f_{c}}$$

where *σ*_max_ and *σ*_min_ denote the maximum and minimum stresses of the fatigue loading, respectively, and *S*_max_ and *S*_min_ represent the maximum and minimum stress levels of the compressive fatigue loading, respectively.

#### 2.2.2. Compressive Fatigue Test

**Figure 1:**
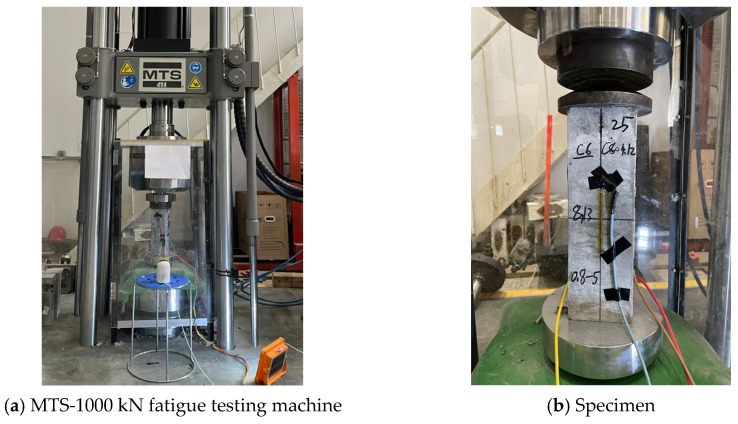
Experimental setup of the fatigue test.

Compressive fatigue tests were conducted on an MTS-1000 kN servo-hydraulic fatigue testing machine (MTS Systems Corporation, Eden Prairie, MN, USA), as shown in Figure 1a, and strain gauges were attached to all four sides at the mid-height of each specimen to monitor axial strain, with a sampling rate of 500 Hz, as shown in Figure 1b. Prior to the fatigue tests, a preload was applied, and the specimens were aligned under compression based on the strain data from all four sides. The specimen end surfaces were carefully prepared to ensure planarity and parallelism, and steel bearing plates were placed at both ends of each specimen. The formal fatigue loading was initiated only when the deviation of the strain on each side from the average value did not exceed 10%. All tests were conducted at room temperature (20 ± 5 °C). The fatigue loading was applied in a stress-controlled mode with constant-amplitude sinusoidal waves at frequencies of 5 Hz or 6 Hz. While an end restraint may introduce certain boundary effects, since all specimens were tested under identical boundary conditions, the influence of such effects on the comparative fatigue behavior was considered limited.

Five groups of maximum stress levels (*S*_max_) were considered in the tests, labeled F-1 to F-5. The fatigue stress levels were defined in a normalized form, where *S*_max_ ranges from 0.70 to 0.90 of the measured compressive strength, and the minimum stress level *S*_min_ is fixed at 0.10. These stress levels do not directly represent service stress conditions but follow established practice in compressive fatigue testing of high-strength concrete [22]. The relatively high maximum stress levels were adopted to enable efficient fatigue testing, while *S*_min_ = 0.10 was used in accordance with standard testing requirements to maintain continuous compressive contact and ensure stable loading conditions. Five specimens were tested in each group, except for the F-5 group (*S*_max_ = 0.70), in which three specimens were tested due to time and economic constraints, as summarized in Table 3. For stress levels of 0.90, 0.85, and 0.80, the application of 6 Hz loading caused the machine to exhibit unstable stress control; therefore, the loading frequency was reduced to 5 Hz to ensure stability. The sampling rate of the strain gauge is 500 Hz.

## 3. Results and Discussion

### 3.1. Axial Compressive Strength

**Figure 2:**
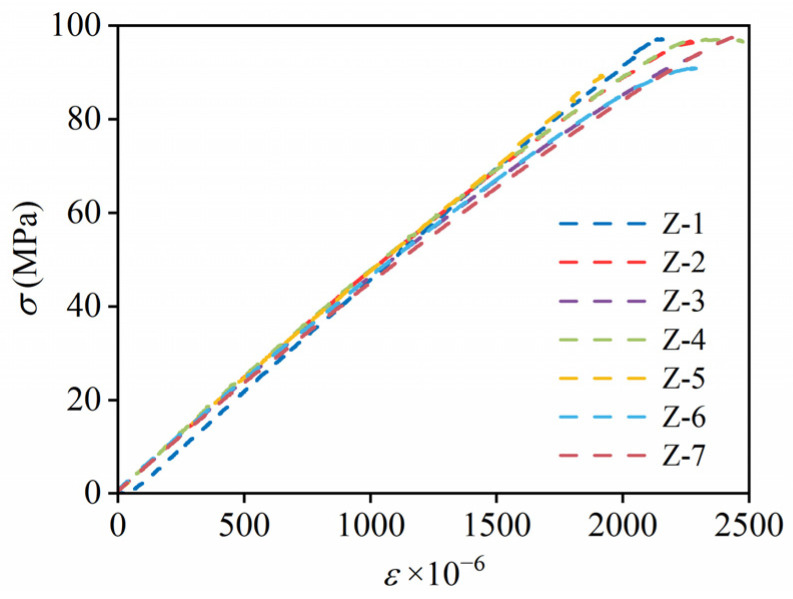
Stress–strain curves of specimens under axial compressive loading.

The average axial compressive strength of the seven specimens cured for 90 days is 94.99 MPa, as shown in Table 4, with a standard deviation of 2.789 MPa and a coefficient of variation (COV) of 0.03; this indicates good consistency between specimens and reliability in the test of axial compressive strength [23]. The elastic modulus is 47.7 GPa, determined from the inclination of the stress–strain curve, as shown in Figure 2. The stress and strain were recorded in real time by the fatigue test machine and the strain gauge, respectively.

### 3.2. Fatigue S–N Curve

**Figure 3:**
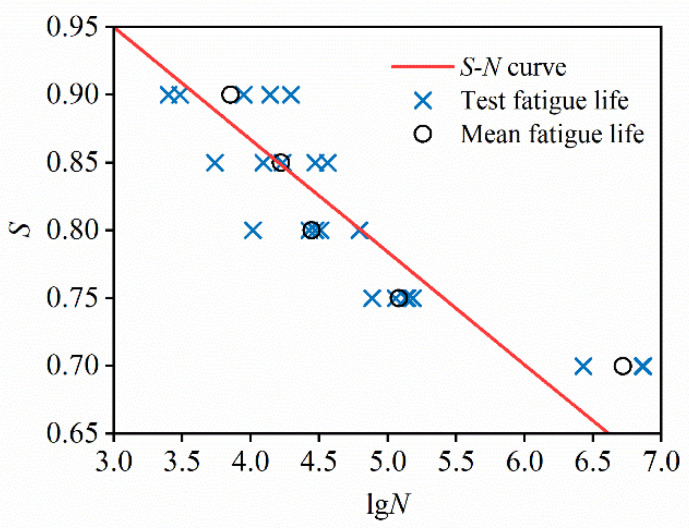
*S–N* curve of C80 concrete under compressive cyclic loading.

The *S–N* curve, which illustrates the relationship between stress level (*S*) and fatigue life (*N*_f_), is a fundamental tool for evaluating the fatigue performance of materials [24]. Based on the results in Table 5, an exponential function is used to fit the experimental data on a semi-logarithmic scale, where *N*_f_ is the average fatigue life corresponding to each stress level. The fitted equation is presented below, with an R-squared (*R*^2^) value of 0.8428, and the corresponding curve is shown in Figure 3.

(3) $$S_{\max}=1.1987-0.0829\mathrm{lg}N_{f}$$

### 3.3. Fatigue Strain Evolution

#### 3.3.1. Three Stages

**Figure 4:**
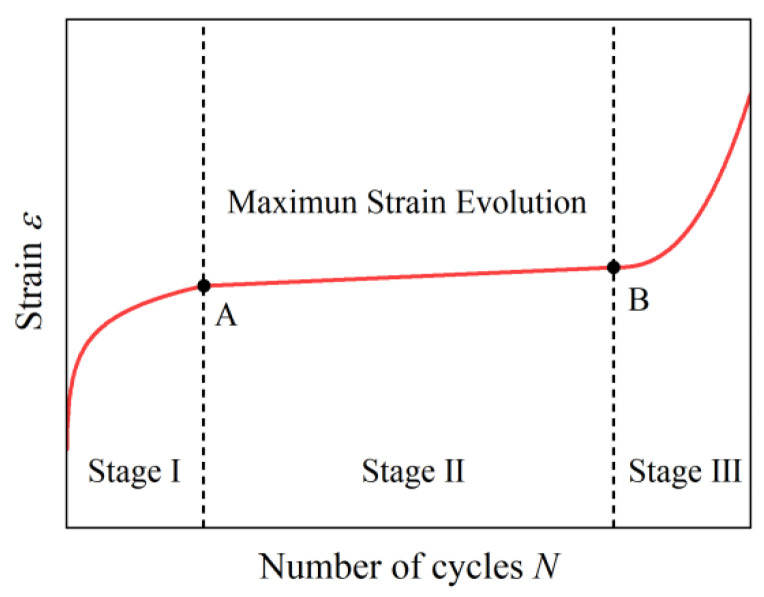
Schematic illustration of three-stage fatigue strain evolution in concrete. Points A and B mark the boundaries of the three stages, whereby point A indicates the beginning of the second stage and point B denotes its end.

Many studies show that the strain evolution of concrete under constant-amplitude cyclic loading typically follows a three-stage pattern [25,26,27,28], as shown in Figure 4, and the fatigue strain is defined as the maximum strain corresponding to the maximum compressive stress within each loading cycle during fatigue testing. For a typical three-stage pattern, the strain increases rapidly in the first stage, which accounts for approximately 60–70% of overall fatigue deformation. The second stage is much longer than the other two stages and is characterized by a linear increase in strain. In addition, fatigue damage accumulation, as well as the evolution of residual strain and stiffness degradation, mainly occurs during the second stage. The third stage is the shortest and involves a sharp acceleration in strain, which ultimately leads to failure. Points A and B mark the boundaries of the three stages, whereby point A indicates the beginning of the second stage and point B denotes its end.

**Figure 5:**
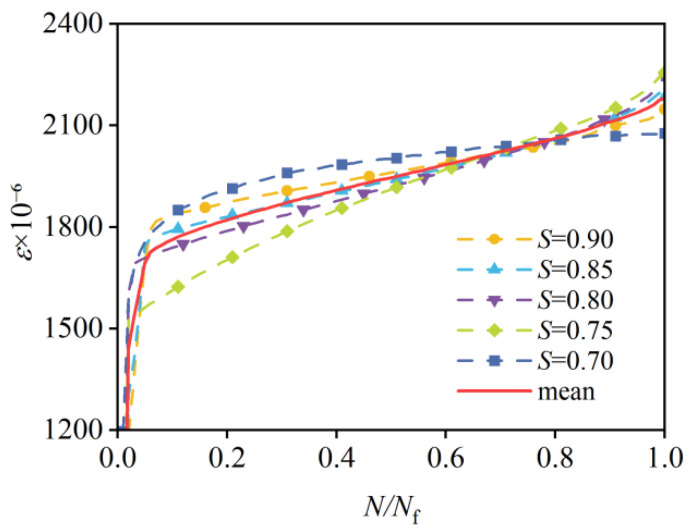
Strain evolution of C80 concrete under compressive fatigue load.

The fatigue strain evolution of C80 concrete was recorded for five stress levels, as shown in Figure 5. It does not exhibit a typical three-stage pattern, since the third stage nearly disappears in the fatigue process. With the decrease in stress level, the initial strain in the second stage also decreases. However, the strain values at the end of the second stage show minor differences among stress levels, and the strain in the second stage exhibits linear variation at all stress levels.

The first stage is very short in duration, accounting for less than 5% of the overall fatigue life. In contrast, for lower-strength concretes (e.g., C20, C30, C35, and C40), the third stage under compressive fatigue load typically spans about 10–15% of the fatigue life [11,16,29]. Previous studies [16,30,31,32] show that in high-strength concrete, the first and third stages are generally less pronounced, each comprising less than 5% of the fatigue life, which is consistent with the findings of this study.

**Figure 6:**
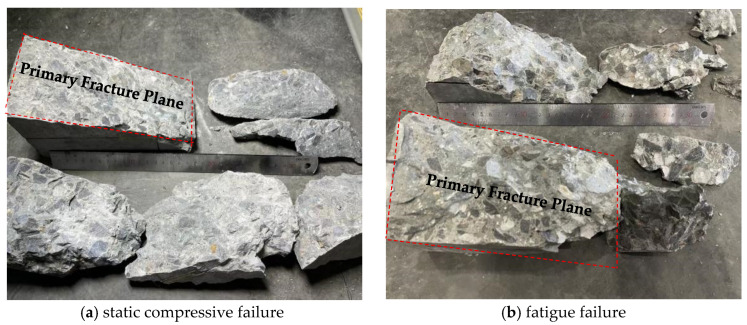
Failure patterns of C80 concrete specimens.

Grade C80 concrete demonstrates extremely brittle behavior in the third stage. As illustrated in Figure 6, the fatigue fracture morphology is remarkably similar to that of static axial compressive failure, with both displaying relatively clean and flat fracture planes. This high degree of similarity indicates quite a limited capacity for plastic deformation, suggesting that the second stage should be the primary focus for fatigue analysis. Such behavior is critical for fatigue life prediction and for the structural design of high-strength concrete components such as wind turbine towers.

#### 3.3.2. Uniqueness of Fatigue Strain

The fatigue failure strain at the end of the second stage ranges from 2079.01 × 10^−6^ to 2180.86 × 10^−6^ for different stress levels, as shown in Table 6, while the static failure strain in the axial compressive strength test is 2293.36 × 10^−6^. The difference between them is 5.0–9.3%, indicating good agreement between the two cases. This difference is 1.3–11.3% for C20 concrete and 0.0–15.0% for C40 concrete, respectively [33]. This phenomenon is consistent with the observed uniqueness of the strain envelope in concrete fatigue [16,29,34], which states that the final strain at the end of the second stage of fatigue (point B in Figure 4) closely approximates the static failure strain, and that the value of the failure strain is the material constant independent of loading history. This implies that fatigue failure in concrete is mainly governed by its intrinsic deformation capacity rather than the specific loading path. Hence, the strain at the end of the second stage serves as a reliable indicator for fatigue failure prediction in high-strength concrete. Moreover, compared with normal-strength concrete, high-strength concrete exhibits smaller variations in fatigue strain at the end of the second stage across stress levels, indicating that as concrete strength and brittleness increase, the second-stage fatigue strain aligns more closely with the static failure strain.

#### 3.3.3. Prediction Model of Fatigue Life

**Figure 7:**
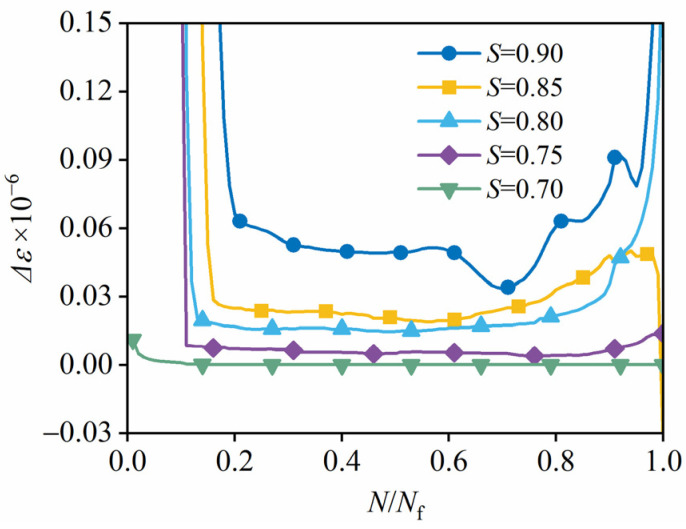
Variation in cyclic strain increments at different stress levels.

Figure 7 illustrates the experimental results on the variation in strain increment under each fatigue loading cycle (Δ*ε*), with respect to the fatigue damage process (*N*/*N*_f_). The strain increment is defined as follows:

(4) $$\Delta \varepsilon =\frac{d\varepsilon }{dN}$$

where *ε* is strain, and *N* is the number of loading cycles.

The increment also follows a three-stage pattern, and the second stage is dominant and relatively stable, showing a linear increase consistent with the analysis in Section 3.3.1. In addition, lower stress levels are characterized by smaller values of strain increase in the second stage, and by smaller percentages for the first and third stages.

The stable linear behavior of the second stage provides a foundation for modeling fatigue life, as evidenced by previous studies. Oneschkow and other researchers [16,35,36,37] have reported a linear relationship between the base-10 logarithm of the cyclic strain increment (Δ*ε*) in the second stage and the base-10 logarithm of fatigue life (*N*_f_).

**Figure 8:**
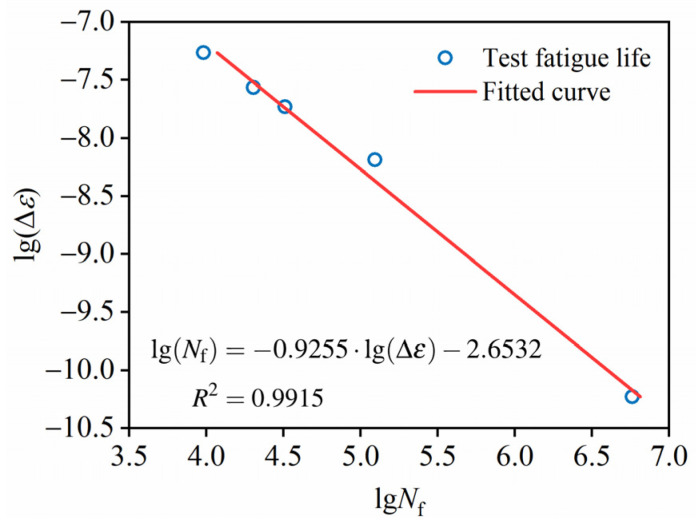
Fitted curve of strain gradient and fatigue life.

This relationship, observed in a log–log plot, indicates a power-law dependence between the strain increment and fatigue life, suggesting that the strain increment can be used as a reliable predictor of fatigue life, forming the basis for a fatigue life prediction model. It can be expressed as follows:

(5) $$\mathrm{lg}(N_{f})=a\cdot \mathrm{lg}(\Delta \varepsilon )+b$$

where *a* and *b* are empirical constants, which are usually determined through experiments. The results of the strain increments in the second stage under five stress levels are shown in Table 7, and constants *a* and *b* are determined to be −0.9255 and 2.6532, respectively. Thus, the prediction model of fatigue life is

(6) $$\mathrm{lg}(N_{f})=-0.9255\cdot \mathrm{lg}(\Delta \varepsilon )-2.6532$$

with an R-squared (*R*^2^) value of 0.9915, which is also illustrated in Figure 8.

#### 3.3.4. Validation of Prediction Model

The fatigue life predicted by the *S–N* curve derived from experimental data in Section 3.2 (denoted by *N_s_*) is compared with that predicted by the strain increment in Section 3.3.3 (denoted by *N_ε_*). The error between the two predictions and the experimental data is calculated as follows:

(7) $$\begin{array}{l} e_{1}=\left|\mathrm{lg}N_{\varepsilon }-\mathrm{lg}N_{f}\right|\\ e_{2}=\left|\mathrm{lg}N_{s}-\mathrm{lg}N_{f}\right| \end{array}$$

where *N*_f_ especially denotes the experimental data on fatigue life.

**Figure 9:**
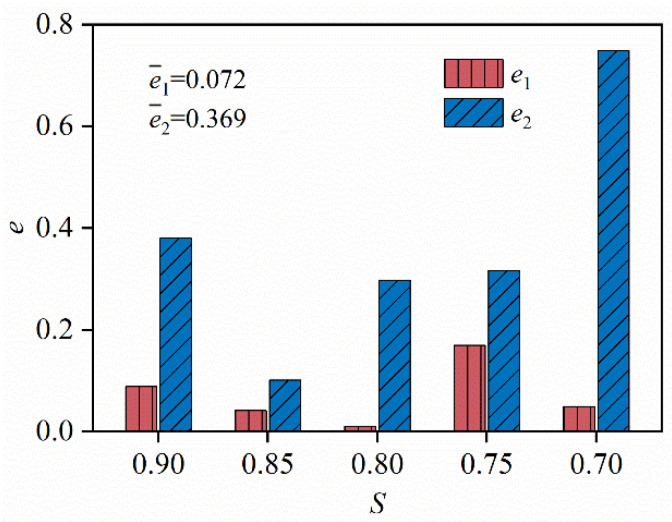
Errors between the two predictions and the experimental data.

Figure 9 shows the derivation of *e*_1_ and *e*_2_ for different stress levels. The fatigue life predicted by the strain-increase rate *N_ε_* shows significantly smaller errors than that predicted by the *S–N* curve for all stress levels. The mean errors of *e*_1_ and *e*_2_ are 0.072 and 0.369, respectively, which indicates that the strain-increase rate represents an excellent model for predicting fatigue life, with much higher accuracy than the *S–N* curve.

### 3.4. Stiffness Degradation Model

#### 3.4.1. Theory of Fatigue Damage

The Palmgren–Miner (P-M) criterion is a classical theory in fatigue analysis based on linear damage accumulation, usually used to quantify accumulative damage in various materials subjected to cyclic loading [38]. The fundamental expression of the P-M criterion is as follows:

(8) $$D=\sum_{i=1}^{n}\frac{N_{i}}{N_{f,i}}$$

where *D* is the index of cumulative fatigue damage, and its value ranges from 0 (no damage) to 1 (fatigue failure). For a fatigue load of varying amplitudes characterized by a series of stress levels *S_i_* (*I* = 1, 2, …, *n*), *N*_f,*I*_ is the fatigue life (i.e., the cyclic number for failure) of the stress level *S_i_*, and *N_i_* is the count of fatigue cycles for the stress level *S_i_*.

For a fatigue load of constant amplitude of a certain stress level, Equation (8) is reduced to

(9) $$D=\frac{N}{N_{f}}$$

where *N* is the count of fatigue cycles, and *N*_f_ is the fatigue life at this corresponding stress level. For a certain count of cycle *N*, the cumulative damage *D* quantitatively denotes the progress of fatigue damage. Accordingly, the fatigue damage increment per cycle is denoted as Δ*D*, given as follows:

(10) $$\Delta D=\frac{1}{N_{f}}$$

#### 3.4.2. Stiffness Degradation in Fatigue Tests

Many studies show that the elastic modulus of concrete gradually decreases under fatigue loading [25,28,39,40,41]. This phenomenon is known as stiffness degradation, which is a critical indicator of cumulative fatigue damage in concrete. In this study, the stiffness degradation of C80 concrete under constant-amplitude fatigue loading is examined, and the secant modulus *E_s_*, defined by the maximum stress *σ*_max_ and maximum strain *ε*_max_ in each cycle, is used to evaluate stiffness degradation:


(11) $$E_{s}=\frac{\sigma_{\max}}{\varepsilon_{\max}}$$

For a certain stress level with a constant *σ*_max_, the secant modulus *E_s_* is negatively correlated with the maximum strain *ε*_max_.

**Figure 10:**
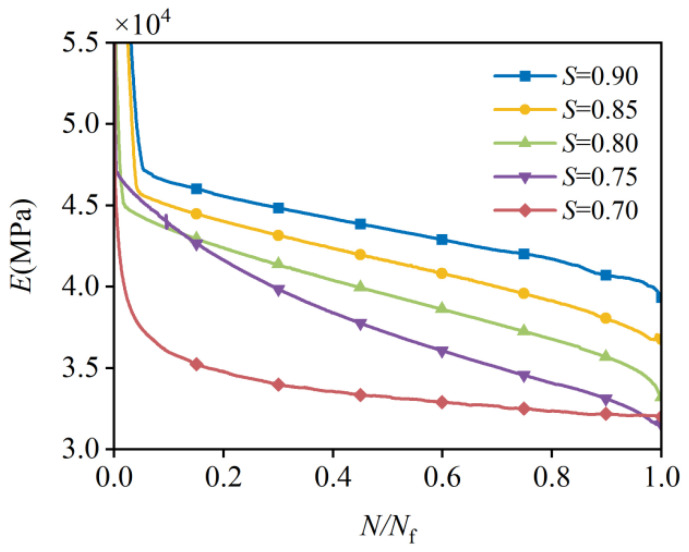
Process of stiffness degradation.

As shown in Figure 10, the stiffness degradation at each stress level corresponds to a decrease in Es throughout the fatigue process, and it exhibits a three-stage pattern similar to that observed in strain evolution. The second stage is characterized by a linear decrease and accounts for more than 90% of the entire fatigue process.

#### 3.4.3. Model of Stiffness Degradation

Based on Continuum Damage Mechanics, Holmam et al. [11,42,43,44] proposed a model to indicate the stiffness degradation of concrete during fatigue damage evolution:

(12) $$E_{s}=E_{0}(1-D)$$

Here, *E*_0_ is initial secant modulus at the beginning of the second stage. Equation (12), although succinct and indicative, reaches an *E_s_* value of zero for cumulative damage *D* = 1, which conflicts with the findings of many experiments; therefore, some modified models are proposed for consistency with existing experimental findings, such as introducing a coefficient k to more accurately describe the damage process [45].

(13) $$E_{s}=E_{0}(1-kD)$$

After calculating Equation (13), the following work is carried out to calibrate coefficient *k* with the experimental data. Then, the change in the secant modulus per loading cycle, denoted as Δ*E_s_*, is calculated by differentiating Equation (13).


(14) $$\Delta E_{s}=\frac{dE_{s}}{dN}=-\frac{dD}{dN}\cdot kE_{0}$$

Since the second stage of the fatigue failure process dominates for all stress levels, the modulus degradation in this stage is used to determine the value of *k*. Based on the experimental data, Table 8 shows the changes in the secant moduli ΔE, as well as the initial secant moduli E0, in the second stage for each stress level.

The term d*D*/d*N* in Equation (14) corresponds to the increase in fatigue damage per cycle and is therefore equal to Δ*D* in Section 3.4


(15) $$\Delta D=\frac{dD}{dN}$$

**Figure 11:**
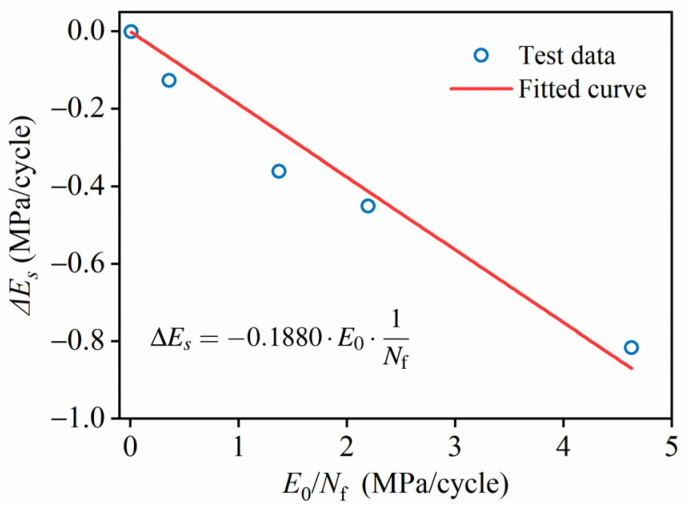
Fitting of factor *k*.

Substituting Equation (15) into Equation (14) gives

(16) $$\Delta E_{s}=-kE_{0}\cdot \Delta D=-kE_{0}\cdot \frac{1}{N_{f}}$$

where *k* is determined by Δ*E_s_*, *E*_0_, and *N*_f_ in Table 8, and the fitting of *k* is shown in Figure 11. Since the slope of the fitted curve is 0.1880, Equation (16) becomes

(17) $$\Delta E_{s}=-0.1880\cdot E_{0}\cdot \frac{1}{N_{f}}$$

where *R*^2^ = 0.9539, indicating good fitting of Equation (17).

Accordingly, the model of stiffness degradation for C80 concrete is


(18) $$E=E_{0}(1-0.1880\cdot D)$$

**Figure 12:**
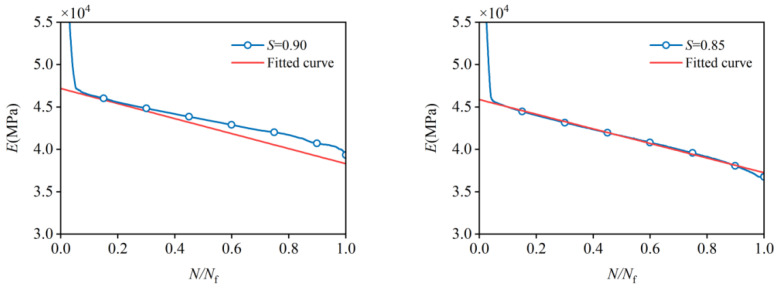

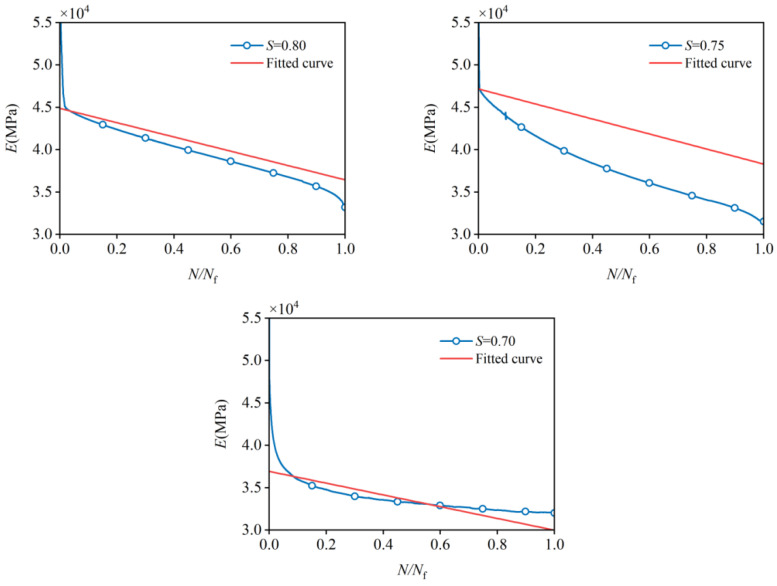
Comparison between experimental and fitted stiffness degradation curves at various stress levels.

Equation (18) is validated against the stiffness degradation in the experiment for all stress levels in Figure 12, and shows excellent agreement for all stress levels. The strong correlation confirms the reliability and applicability of Equation (18), providing a theoretical basis for numerical simulations of concrete fatigue using finite element methods, as well as practical guidance for structural design in engineering applications.

### 3.5. Limitations of the Models

Although the proposed empirical models for fatigue life (Equation (6)) and stiffness degradation (Equation (13)) are based on experimental data and demonstrate high predictive accuracy (R^2^ > 0.90), certain limitations should be acknowledged when applying them to practical structural design or numerical simulations:

(1) Loading Conditions: The models were developed based on constant-amplitude uniaxial compression. In actual wind turbine towers, concrete is often subjected to variable-amplitude loading and multi-axial stress states, which may affect the damage accumulation rate.
(2) Environmental Factors: The experiments were conducted at room temperature. As such, the coupled effects of moisture, temperature fluctuations, and chemical erosion common in offshore environments were not accounted for.
(3) Material Specificity: The empirical constants were calibrated specifically for the C80 mix, so their generalizability to other concrete grades or those with fiber reinforcements requires further experimental validation.

## 4. Conclusions

Based on the experimental results and corresponding analysis, the following main conclusions can be drawn.

(1) C80 concrete exhibits high static strength and stiffness, confirming its excellent material stability and suitability for applications in high-stress, high-fatigue environments such as ultra-high wind turbine towers.
(2) The strain evolution follows a distinct three-stage pattern, where the second stage, which has a long duration, reflects a steady accumulation of internal microcracks. This stage dominates fatigue life and controls failure evolution, indicating that fatigue failure is essentially governed by progressive microcrack coalescence rather than sudden instability. Therefore, the second-stage evolution law can be used to predict the fatigue life and cumulative damage of C80 concrete.
(3) The maximum strain at the end of the second fatigue stage under different maximum stress levels approaches the ultimate static strain, which indicates that once the critical strain is reached, the concrete undergoes rapid fatigue failure regardless of the rate of fatigue accumulation, revealing a deformation-controlled failure mechanism.
(4) Based on the linear increase in strain during the second fatigue stage, a fatigue life prediction model is developed using cyclic strain increments. The model produces an average error of 0.072, which is significantly lower than the average error of 0.369 produced by the traditional *S–N* curve, demonstrating superior prediction accuracy. This model enables fatigue life prediction based on real-time strain measurements from engineering monitoring, providing a theoretical approach for the real-time assessment of fatigue performance in wind turbine towers.
(5) At the end of the second fatigue stage, C80 concrete retains relatively high stiffness, exhibiting the characteristic stiffness degradation behavior of high-strength concrete under fatigue loading. By integrating the experimental data with the Palmgren–Miner damage accumulation rule, a stiffness degradation model for C80 concrete is established, which accurately reproduces the stiffness evolution under fatigue loading and provides a reliable basis for finite element-based fatigue damage simulations, thereby supporting the optimization of structural design under high-cycle fatigue conditions.

Several limitations of this study should be acknowledged. First, this study primarily focuses on the macroscopic phenomenological response of C80 concrete. Due to the absence of microstructural characterization (e.g., SEM, XRD, or porosity analysis), the specific influence of mineral phases (such as C_3_S and C_3_A) and of the interfacial transition zone (ITZ) on the fatigue degradation process needs to be further elucidated. Second, the proposed fatigue life and stiffness degradation models are empirically calibrated based on the specific concrete mix design used in this study (P-II 52.5 cement and crushed aggregate). Therefore, the generalizability of these model parameters to other C80 compositions requires further validation with broader material databases. Furthermore, the present study focuses on uniaxial constant-amplitude fatigue loading, without considering the effects of variable-amplitude or multiaxial stress states that commonly occur in practical wind turbine tower structures. The proposed strain- and stiffness-based models describe the linear degradation stage effectively, but the nonlinear behavior near failure and the influence of cyclic plasticity need to be further examined. Future research should bridge the gap between micro-mechanisms and macro-performance by integrating experimental monitoring techniques such as acoustic emission or X-ray CT scanning, and extend the developed models to multiaxial and structural-scale simulations, to improve fatigue assessment of large-scale concrete wind turbine towers.

## Figures and Tables

**Table 1:** C80 high-strength concrete mixtures (kg/m^3^).

Cement	Fly Ash	GGBS	Sand	Coarse Aggregate	Admixture	Water	w/b
435	25	105	760	1050	9.2	145	0.26

**Table 2:** Specimen dimensions and quantities.

Test Type	Specimen ID	Dimensions	Number
Axial compressive test	Z-1~Z-7	100 mm × 100 mm × 300 mm	7
Compressive fatigue test	F-1-1~F-5-3	100 mm × 100 mm × 300 mm	23

**Table 3:** Loading conditions for compressive fatigue experiment.

Test Group	Maximum Stress Level	Minimum Stress Level	Strain Gauge Sampling Frequency (Hz)	Loading Frequency (Hz)	Number of Specimens
F-1	0.90	0.1	500	5	5
F-2	0.85	0.1	500	5	5
F-3	0.80	0.1	500	5	5
F-4	0.75	0.1	500	6	5
F-5	0.70	0.1	500	6	3

**Table 4:** Axial compressive strength test results.

	Items	*f*_c_
Specimen ID	Z-1	97.17 MPa
	Z-2	96.68 MPa
	Z-3	94.35 MPa
	Z-4	97.04 MPa
	Z-5	90.71 MPa
	Z-6	91.08 MPa
	Z-7	97.91 MPa
Statistical data	Mean *f*_c_ (MPa)	94.99 MPa
	Standard value *f*_ck_ (MPa)	90.40 MPa
	Standard deviation	2.79 MPa
	Coefficient of variation (COV)	0.03

**Table 5:** Fatigue life under compressive cyclic loading.

Stress Level (*S*)	0.90(F-1)	0.85(F-2)	0.80(F-3)	0.75(F-4)	0.70(F-5)
Specimen 1	2514	36,589	62,300	136,514	2,691,097
Specimen 2	13,876	12,349	29,759	153,320	7,370,025
Specimen 3	19,634	17,083	32,303	139,666	7,303,485
Specimen 4	3050	29,701	10,425	114,547	—
Specimen 5	8930	5495	26,872	77,229	—
Mean fatigue life *N*_f_	9600.8	20,243.4	32,331.8	124,255.2	5,788,202.3
Standard deviation	6519.6	11,370.6	16,819.2	26,601.7	2,190,152.7
Coefficient of variation	0.679	0.562	0.520	0.214	0.378

**Table 6:** Fatigue strain at the end of the second stage for concretes of different strengths.

Concrete Grade	Concrete Strength (MPa)	Stress Level	Failure Strain (×10^−6^)	Difference (%)
C80	81.3	Static	2293.36	—
		0.90	2105.70	8.2
		0.85	2156.55	6.0
		0.80	2147.18	6.4
		0.75	2180.86	5.0
		0.70	2079.01	9.3
C20 [33]	23.7	Static	1500	—
		0.89	1480	1.3
		0.76	1350	10.0
		0.68	1330	11.3
C40 [33]	43.3	Static	2000	—
		0.65	2300	15.0
		0.62	2150	7.5
		0.58	2000	0.0

**Table 7:** Strain increment (Δε) in the second stage under different stress levels.

**Stress Level (*S*)**	0.90	0.85	0.80	0.75	0.70
**Strain Increment Δ*ε* (×10^−8^)**	5.43	2.73	1.87	0.65	0.00592

**Table 8:** Changes in secant moduli in stage II of stiffness degradation at different stress levels.

**Stress Level**	0.90	0.85	0.80	0.75	0.70
***E*_0_ (MPa)**	47,188.3	45,882.0	44,879.4	47,173.5	36,928.4
**Δ*E*_s_ (MPa/Per cycle)**	−0.81630	−0.45094	−0.36134	−0.12604	−0.00094
***N*** **_f_**	9600.8	20,243.4	32,331.8	124,255.2	5,788,202.3

## Data Availability

The original contributions presented in this study are included in the article. Further inquiries can be directed to the corresponding author.

## Nomenclature

*f*_c_	Axial compressive strength
*f*_ck_	The standard value of axial compressive strength
*σ*_max_ and *σ*_min_	The maximum and minimum stresses of the fatigue loading
*S*_max_ and *S*_min_	The maximum and minimum stress levels of the compressive fatigue loading
*N*	The number of fatigue cycles
*N*_f_	The average fatigue life corresponding to each stress level
*N*_ε_	The fatigue life predicted by the strain increment
*N*_s_	The fatigue life predicted by the *S–N* curve
*ε*	Strain
Δ*ε*	The strain increment under each fatigue loading cycle
*D*	The index of cumulative fatigue damage
Δ*D*	The fatigue damage increment per cycle
*E*_s_	The secant modulus
*E*_0_	The initial secant modulus at the beginning of the second fatigue loading stage
Δ*E*_s_	The secant modulus variation per loading cycle
*e*_1_ and *e*_2_	The error between the two predictions and the experimental data
*R* ^2^	The coefficient of determination
